# Benchmarking of Large Language Models for the Dental Admission Test

**DOI:** 10.34133/hds.0250

**Published:** 2025-04-01

**Authors:** Yu Hou, Jay Patel, Liya Dai, Emily Zhang, Yang Liu, Zaifu Zhan, Pooja Gangwani, Rui Zhang

**Affiliations:** ^1^ Division of Computational Health Sciences, Department of Surgery, University of Minnesota, Minneapolis, MN 55455, USA.; ^2^ Center for Learning Health System Sciences, University of Minnesota, Minneapolis, MN 55455, USA.; ^3^ Kornberg School of Dentistry, Oral Health Sciences Department, Temple University, Philadelphia, PA 19140, USA.; ^4^ Wayzata High School, Plymouth, MN 55446, USA.; ^5^ School of Education, Syracuse University, Syracuse, NY 13244, USA.; ^6^ Department of Electrical and Computer Engineering, University of Minnesota, Minneapolis, MN, USA.

## Abstract

**Background:** Large language models (LLMs) have shown promise in educational applications, but their performance on high-stakes admissions tests, such as the Dental Admission Test (DAT), remains unclear. Understanding the capabilities and limitations of these models is critical for determining their suitability in test preparation. **Methods:** This study evaluated the ability of 16 LLMs, including general-purpose models (e.g., GPT-3.5, GPT-4, GPT-4o, GPT-o1, Google’s Bard, mistral-large, and Claude), domain-specific fine-tuned models (e.g., DentalGPT, MedGPT, and BioGPT), and open-source models (e.g., Llama2-7B, Llama2-13B, Llama2-70B, Llama3-8B, and Llama3-70B), to answer questions from a sample DAT. Quantitative analysis was performed to assess model accuracy in different sections, and qualitative thematic analysis by subject matter experts examined specific challenges encountered by the models. **Results:** GPT-4o and GPT-o1 outperformed others in text-based questions assessing knowledge and comprehension, with GPT-o1 achieving perfect scores in the natural sciences (NS) and reading comprehension (RC) sections. Open-source models such as Llama3-70B also performed competitively in RC tasks. However, all models, including GPT-4o, struggled substantially with perceptual ability (PA) items, highlighting a persistent limitation in handling image-based tasks requiring visual-spatial reasoning. Fine-tuned medical models (e.g., DentalGPT, MedGPT, and BioGPT) demonstrated moderate success in text-based tasks but underperformed in areas requiring critical thinking and reasoning. Thematic analysis identified key challenges, including difficulties with stepwise problem-solving, transferring knowledge, comprehending intricate questions, and hallucinations, particularly on advanced items. **Conclusions:** While LLMs show potential for reinforcing factual knowledge and supporting learners, their limitations in handling higher-order cognitive tasks and image-based reasoning underscore the need for judicious integration with instructor-led guidance and targeted practice. This study provides valuable insights into the capabilities and limitations of current LLMs in preparing prospective dental students and highlights pathways for future innovations to improve performance across all cognitive skills assessed by the DAT.

## Introduction

The Dental Admission Test (DAT) is widely used as a crucial metric by many educational institutions to evaluate applicants, with numerous studies indicating a positive correlation between the DAT scores and students’ performance post-admission [1,2]. In recent years, large language models (LLMs) have emerged as valuable tools across diverse domains, including healthcare education. Applications of LLMs in healthcare education encompass personalized learning, interactive tutoring, case-based learning, and patient simulation [3]. However, ensuring the accuracy and reliability of LLMs in healthcare education is paramount to engender trust and confidence among healthcare educators, students, administration, and other stakeholders, as well as to mitigate bias [4].

Despite some studies exploring the performance of LLMs on various medical exams [5–7], there has been limited investigation on the integration of LLMs into dental admission assessment, which hinders the understanding of how to effectively leverage LLMs to support students’ learning in this specific domain. Therefore, it is imperative to evaluate the performance of LLMs on the DAT and reveal the potential and limitations of using LLMs in the DAT preparation. Our examination aims to contribute to the ongoing exploration of LLMs in healthcare education, shedding light on their potential utility and effectiveness within the realm of dental education. Through rigorous testing and validation, our study endeavors to provide insights into the suitability of LLMs for enhancing assessment processes in dental education, thereby facilitating informed decision-making and advancing educational practices in dentistry.

The present study evaluated the performance of 16 LLMs, including popular general-purpose models (GPT-3.5 [8], GPT-4 [9], GPT-4o [10], GPT-o1 [11], Google’s Bard [12], mistral-large [13], and Claude [14]), domain-specific fine-tuned models (MMedC [15], DentalGPT [16], MedGPT [17], and BioGPT [18]), and open-source models (Llama2-7B [19], Llama2-13B [20], Llama2-70B [21], Llama3-8B [22], and Llama3-70B [23]), on the DAT to address the following research questions: (a) How do different LLMs perform when taking the DAT? (b) What are the potentials and limitations of using these LLMs in the DAT preparation?

## Methods

### Dataset

The DAT is a computer-based exam accepted by numerous dental schools across the United States and Canada, totaling 66 and 10 institutions, respectively [24]. Designed to assess applicants’ potential for success, the DAT covers various domains, including natural sciences (NS), perceptual ability (PA), reading comprehension (RC), and quantitative reasoning (QR). In this study, a publicly available DAT sample test was obtained from the American Dental Association (https://www.ada.org/). This sample test comprises 247 multiple-choice questions distributed across the four distinct testing components. These components collectively represent the breadth of knowledge and skills required for success in dental education and practice.

### Method

We employed 16 LLMs: GPT-3.5, GPT-4, GPT-4o, GPT-o1, Google’s Bard, mistral-large, Claude, MMedC, DentalGPT, MedGPT, BioGPT, Llama2-7B, Llama2-13B, Llama2-70B, Llama3-8B, and Llama3-70B. All models were tested with text-based questions, while GPT-4o, GPT-o1, Bard, Claude, DentalGPT, MedGPT, and BioGPT were also evaluated on image-based questions. Notably, the PA component consisted exclusively of image-based questions and answers. Additionally, certain sections, such as the QR section, included geometric problems that required image-based presentations due to formatting constraints. Because GPT-3.5, GPT-4, MMedC, Llama models, and Mistral could not process images directly, we captured screenshots of the image-based questions and forwarded them to Bard, GPT-4o, GPT-o1, Claude, DentalGPT, MedGPT, and BioGPT for processing. Specific prompts tailored for each test section are described below.

All RC questions are text-based. Each question was presented as a separate chat to each LLM. The chat began with the main prompt: “This is a simulated Dental Admission Test. We need an accurate answer. Only one choice is the correct answer. Please tell us which one of the choices is the correct answer and give a SHORT explanation (less than 4 sentences) on why it is correct.” Immediately following the main prompt, a text-based question was submitted to the testing model for its answer and explanation. This process was repeated for all RC questions across all the LLMs.

For the PA questions, the DAT includes special instructions for different types of perceptual questions. After entering the main prompt, the original text instructions from the DAT were submitted to models. Most questions in the NS and QR sections are text-based. Therefore, they were processed using the same method as the RC questions. For image-based NS and QR questions, we applied the same method used for the PA questions, where screenshots of the image-based questions were provided to GBard, GPT-4o, GPT-o1, Claude, DentalGPT, MedGPT, and BioGPT for processing.

### Evaluation

To assess the performance and explanatory capabilities of the 16 LLMs on the DAT questions, a comprehensive evaluation process was conducted, encompassing both quantitative and qualitative analyses. Two types of data were evaluated: The answer options provided by the models and the model-generated texts (answer justifications). For the quantitative analysis, the performance of LLMs was assessed by calculating the percentage of correct answers provided by each of the seven models across all items. This step helped determine the accuracy of the LLMs’ answer selections.

The qualitative analysis focused on the model-generated texts. Two subject matter experts (SMEs) conducted a thematic analysis to evaluate the explanations provided by the LLMs. Both SMEs are faculties in disciplines related to dental or oral health. A stratified random sampling approach was employed to ensure a representative sample, grouping items by format (text-based and image-based), test components, and model correctness. Fifty items were randomly selected from the test sample, resulting in a sample that included 72 text-based items and 26 image-based questions, totaling 98 justifications for the selected 50 questions. The SMEs first analyzed the text data for accuracy and concordance in the explanations. Accuracy was defined as whether a model correctly understood a question and provided a supporting explanation. An explanation could be deemed inaccurate due to incorrect information, logical fallacies, or statistical errors. Concordance referred to the coherence and soundness of the argument presented in the explanation. To synthesize the text data, the SMEs initially analyzed model-generated texts for 10 questions to identify underlying themes. They then discussed their findings and consolidated the identified themes into seven categories. Subsequently, they reanalyzed the texts for these 10 questions, assigning each explanation to one of the seven categories, achieving a high level of agreement in their assignments (Kappa = 0.96). Given the high level of inter-rater reliability, they divided and separately assessed the remaining text data.

## Results

### Test performance of 16 LLMs

**Fig. 1.**
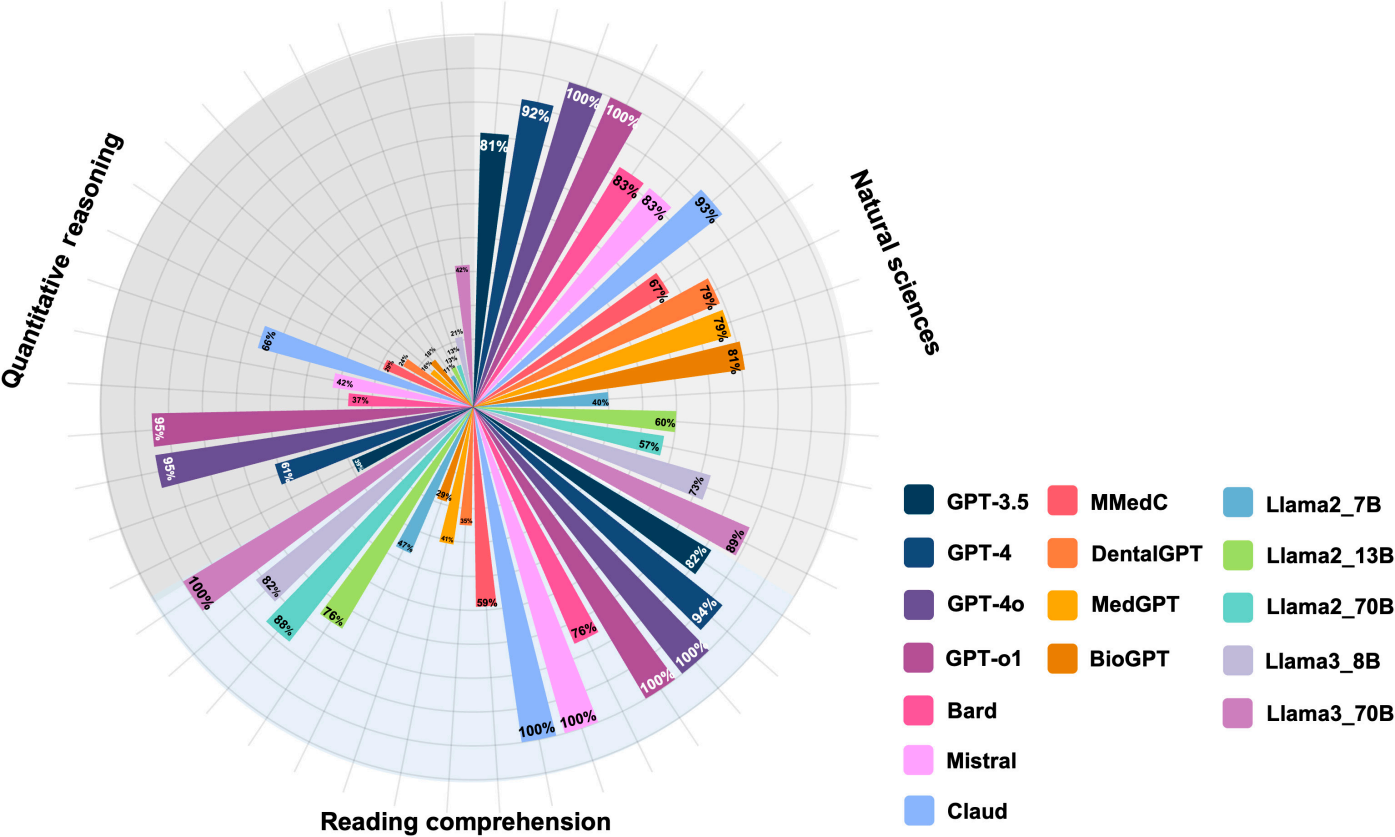
Percentage of correct answers for the text-based test part by different models.

**Fig. 2.**
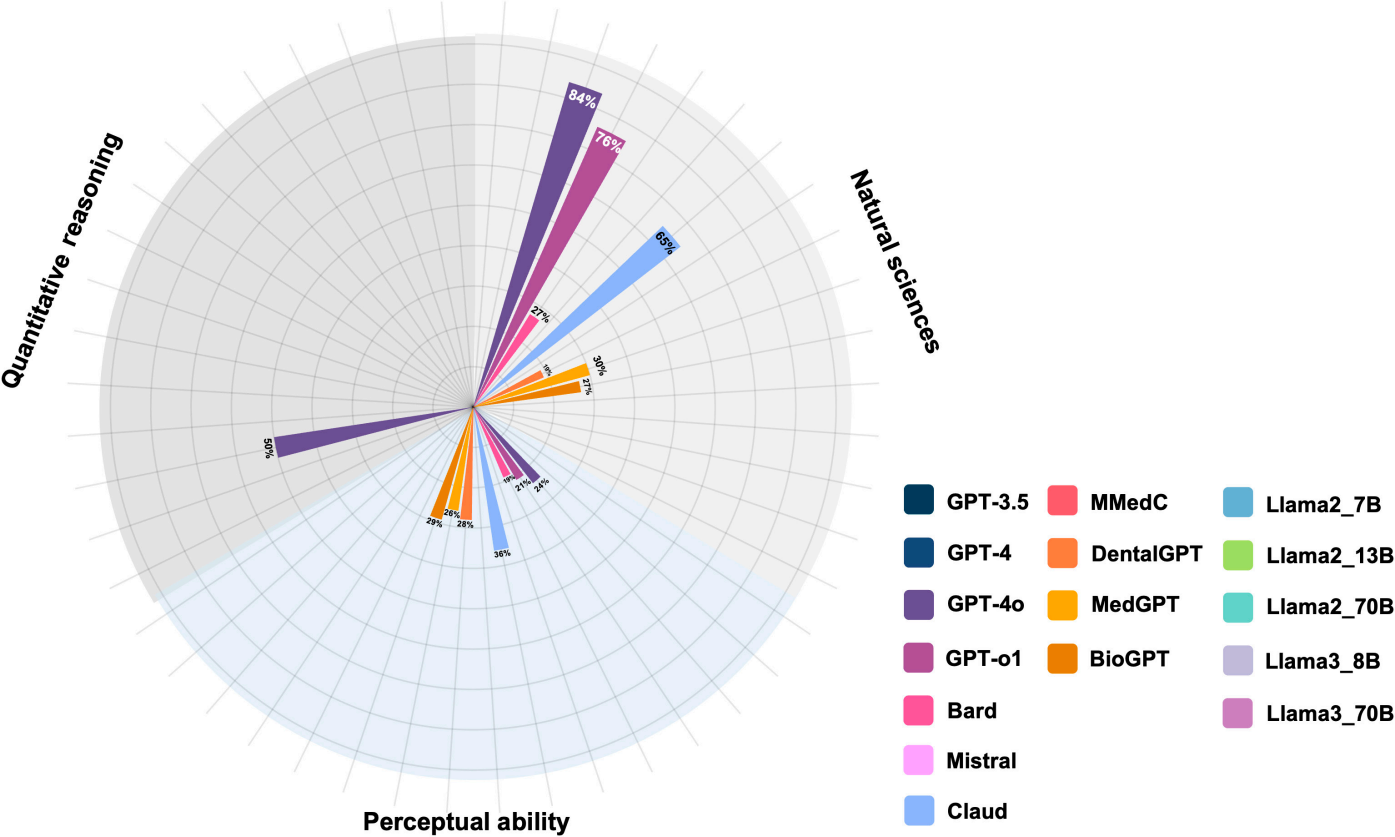
Percentage of correct answers for the image-based test part by different models.

Figures 1 and 2 illustrate the relative performance of the 16 LLMs tested in this study. Overall, commonly used LLMs such as GPT-4o, GPT-3.5, Bard, Claude, and GPT-o1 demonstrated strong performance on the NS and RC sections, with accuracy rates generally exceeding 80%. Among these, GPT-4o achieved the highest performance across all text-based sections, excelling in NS (100%), RC (100%), and QR (95%), indicating its robustness in handling text-based questions. Similarly, GPT-o1 also showed exceptional performance with perfect accuracy in NS (100%) and RC (100%) and a strong result in QR (95%). The performance of domain-specific fine-tuned models such as DentalGPT, MedGPT, and BioGPT varied. While these models demonstrated moderate accuracy in the NS section (DentalGPT: 79%, MedGPT: 79%, BioGPT: 81%), their performance in the RC and QR sections was weaker, particularly in QR (DentalGPT: 24%, MedGPT: 16%, BioGPT: 18%). The open-source models, particularly Llama3-70B, showed competitive performance, achieving 89% accuracy in the NS section and 100% in RC, outperforming several other models in these areas. However, other open-source models like Llama2-7B and Llama2-13B exhibited relatively lower performance, with NS accuracy rates of 40% and 60%, respectively, and RC accuracy rates of 47% and 76%.

The ability of LLMs to handle image-based questions remained limited. GPT-4o, Bard, and Claude showed significantly weaker performance in the PA section, with accuracy rates of 24%, 19%, and 36%, respectively. Similarly, GPT-o1 achieved only 21% in PA. The domain-specific fine-tuned models (DentalGPT, MedGPT, BioGPT) also struggled with image-based PA questions, with accuracy rates of 28%, 26%, and 29%, respectively. Interestingly, the NS section involving image-based questions further highlighted these differences. GPT-4o achieved a relatively strong performance (84%), while the fine-tuned models exhibited lower accuracy (DentalGPT: 19%, MedGPT: 30%, BioGPT: 27%). However, the open-source Llama models were not tested for these image-based tasks, leaving a gap in their evaluation.

### Performance of 16 LLMs on model-generated texts

The SMEs identified seven themes of errors made by the LLMs via the evaluation of the model-generated texts. The themes include (a) critical thinking, (b) stepwise analysis, (c) transfer knowledge to the correct answer, (d) intricate questions, (e) hallucinations, (f) unnecessary complexity, and (g) elimination process. See Table 1 for descriptions of the seven themes.

**Table 1.** Themes of errors made by LLMs

Themes of the LLM’s error performance	Description
Critical thinking	Critical thinking requires the examinees to be able to interpret, analyze, evaluate, explain, and conclude the problems that exist. The models’ performance deteriorated when critical thinking was involved.
Stepwise analysis	The models did not perform well with questions involving stepwise thinking. For example, three steps might be included to get the right answer. The models would get one or two steps fine and then perform wrongly on the next.
Transfer knowledge to the correct answer	The models would do the right calculations; however, they would not report the correct answers on some occasions.
Intricate questions	The models could not understand complex questions. These questions were misinterpreted by the models, leading to false answers.
Hallucinations	In some cases, the models provided completely wrong information, and the source of the information is unsure. This was observed when solving complex problems.
Unnecessary complexity	The models added some unnecessary complexity to the math calculation.
Elimination process	The models failed to use an option elimination process for the perceptual ability and math sections.

Thematic analysis revealed that while the LLMs could answer basic factual science questions that primarily assess factual knowledge retention, their performance declined considerably when critical thinking skills were necessary. These critical thinking questions required more than simple knowledge recall; models had to process information based on logical reasoning, make judgments, and draw conclusions. Additionally, the models struggled with basic science questions involving stepwise analysis, where multiple steps were required, and the output of each step served as input for the subsequent one.

Furthermore, the models faced challenges in comprehending intricate, complex questions, often misinterpreting them and providing incorrect answers. In some instances, the models generated completely erroneous information, potentially hallucinating responses from uncertain sources when solving complex problems. While the Bard model effectively utilized an elimination process for basic science questions, it failed to apply this strategy consistently across other sections, such as PA and mathematics. The analysis also highlighted instances where models added unnecessary complexity to mathematical calculations or failed to report the correct final answer despite performing accurate intermediate calculations. Table 2 shows seven examples of SMEs’ evaluations of the LLMs’ sample questions.

**Table 2.** Examples of SMEs’ evaluations of model-generated texts for the sample questions

Themes	Sample questions (correct answers are bolded)	Model response	SME comments
Critical thinking	In which reaction is H2O considered to be acting as an acid? A. Zn(s) + 2H3O+→Zn2+ + H2(g) + H2O B. HCl(g) + H2O→H3O+ + Cl- C. HC2H3O2 + H2O⇔H3O+ +C2H3O2- **D. NH3 + H2O**⇔**NH4+ + OH-** E. NH3 + H3O+→NH4+ + H2O	Answer: C. Explanation: In this reaction, water donates a proton to the acetic acid molecule, which is a proton acceptor. This makes water an acid, according to the Bronsted-Lowry definition of acids and bases. The other reactions do not involve water donating a proton.	The program knows facts and background. Chemical equations require knowing the topic really well. They are not facts. The model can’t think through to come to the right answer.
Stepwise thinking	A mother’s age is three times her daughter’s age. In twelve years, the mother’s age will be twice the daughter’s age at that time. How old is the mother now? A. 18 B. 20 C. 24 D. 30 **E. 36**	Answer: A. Explanation: Let’s denote the daughter’s current age as x and the mother’s current age as 3x. According to the problem, in 12 years, the mother will be twice as old as the daughter. We can express this in the equation: 3x + 12 = 2 * (x + 12). Solving this equation, we find that x = 6, which means the mother’s current age is 3x = 18 * 3 = 30 years.	The model got the right steps but did wrong calculations after the second step.
Transfer knowledge to the correct answer	Which of the following fractions is the smallest? **A. 11/15** B. 4/5 C. 21/25 D. 5/6 E. 13/17	Answer: E. Explanation: In decimal form: A. 11/15 = 0.7333; B. 4/5 = 0.8; C. 21/25 = 0.84; D. 5/6 = 0.8333; E. 13/17 = 0.7647	The model did the right calculations but picked up the wrong answer.
Inability to understand convoluted questions	A painting which is 4 feet wide and 5 feet long is surrounded by a rectangular frame 6 inches wide. What percent of the area of the picture and the frame is occupied by the frame? A. 10 1/2 B. 20 C. 30 **D. 33 1/3** E. 50	Answer: C. Explanation: The area of the painting is 4 * 5 = 20 square feet. The area of the frame is 6 * 12 = 72 square inches = 1.2 square feet. The total area of the picture and the frame is 20 + 1.2 = 21.2 square feet. The percentage of the area of the picture and the frame that is occupied by the frame is 1.2 / 21.2 * 100 = 5.625 * 100 = 30%.	The model did not fully understand the question.
Hallucinations	Of the following, which group of invertebrates is apparently most closely related to primitive vertebrates? A. Annelida B. Mollusca C. Cnidaria D. Arthropoda **E. Echinodermata**	Answer: C. Explanation: Among the given choices, Cnidaria (jellyfish, corals, and sea anemones) is the group of invertebrates that is most closely related to primitive vertebrates. Cnidarians and primitive vertebrates share a common ancestor and belong to the same lineage, known as the Eumetazoa. Both groups exhibit tissue-level organization and have a rudimentary nervous system, suggesting a closer evolutionary relationship.	The model used the wrong information.
Unnecessary complexity	If [b (c + d) + e]a = 135, then which variable cannot be zero? **A. a** B. b C. c D. d E. e	Answer B. Explanation: If b were equal to 0, then the expression [b (c + d) + e]a would be equal to 0, regardless of the values of c, d, and e. However, we are given that the expression [b (c + d) + e]a is equal to 135. Therefore, b cannot be equal to 0. The other variables, a, c, and d, could be equal to 0 without affecting the value of the expression.	A simple rule - the denominator can’t be zero - can solve the problem.
Elimination process	If 2x - 3 > 3x + 7, then which must be true? A. x > -4 B. x > 4 C. x > -10 D. x < -4 **E. x** < **-10**	Answer: D. Explanation: The answer is D. x < -4. First, we simplify the inequality 2x - 3 > 3x + 7. This gives us -5 > x. We then negate the inequality and multiply both sides by -1. This gives us x < -5. Finally, we look at the answer choices and see that only choice D, x < -4, is consistent with the inequality x < -5. The other choices are incorrect because they do not satisfy the given inequality. For example, choice A, x > -4, would imply that x is greater than -4, which is not true given the inequality x < -5.	The BART model often uses an option elimination process. However, it failed to get the right answer in many cases.

### Potentials and limitations of using LLMs in DAT preparation

According to the quantitative and qualitative results, we further examined the potential and limitations of using the LLMs in the DAT preparation following Bloom’s Taxonomy. Bloom’s Taxonomy is a hierarchical framework that categorizes different levels of cognitive skills and learning objectives [24]. Developed by Benjamin Bloom and his colleagues in the 1950s, the taxonomy is widely used in educational settings to design curricula and assessments [25]. The taxonomy consists of six levels, and the levels progress from simple knowledge and comprehension to more complex levels of application, analysis, synthesis, and evaluation.

From the perspective of Bloom’s Taxonomy, the DAT aims to assess various cognitive levels to evaluate a prospective dental student’s academic abilities and potential. The taxonomy provides a framework for understanding the different levels of cognitive skills and knowledge that the DAT may target.

Knowledge: Some questions in NS assess the foundational knowledge of applicants in areas such as biology, general chemistry, and organic chemistry. This level of the taxonomy involves recalling and recognizing factual information essential for dental education.

Comprehension: Most RC questions evaluate applicants’ ability to understand and interpret information presented in different formats, such as RC passages or data interpretation questions. This level requires understanding the meaning of concepts and demonstrating an understanding of the material.

Application: The DAT includes questions, specifically in the NS and QR sections, that require applying learned concepts, principles, and theories to solve problems or analyze situations related to dental science and practice. This level assesses the ability to apply knowledge in practical contexts.

Analysis: The test includes questions, particularly in the PA section, that assess analytical skills, such as breaking down complex information, identifying relationships, and recognizing patterns or trends. This level evaluates the ability to critically analyze and interpret data relevant to dental practice.

Synthesis: Some questions on the DAT, such as those in the RC and NS sections, require synthesizing information from various sources or combining different concepts to formulate solutions. This level assesses the ability to integrate knowledge and generate original thoughts or hypotheses.

Evaluation: Some DAT questions (e.g., NS and QR questions) require evaluating and making judgments based on specific criteria or standards. This level assesses the ability to critically evaluate information, ideas, or solutions and provide justifications or critiques.

In this study, all LLMs demonstrated promising capabilities in understanding and answering text-based questions, particularly those involving RC and factual knowledge. The study found that LLMs like GPT-4, GPT-4o, GPT-o1, and Claude outperformed other models in accurately responding to text-based items in areas such as NS and RC. This potential could be leveraged in the DAT preparation by using LLMs to explain and reinforce concepts, provide practice questions, and offer targeted feedback for text-based sections of the exam. Moreover, the ability of LLMs to engage in conversational interactions allows for a more interactive and personalized learning experience. Students could ask follow-up questions, seek clarifications, and receive tailored explanations from the LLMs, potentially enhancing their understanding of complex topics and improving their critical thinking skills.

While LLMs excel in text-based tasks, the study revealed significant limitations in their ability to solve QR and PA problems. The thematic analysis results suggested that most LLMs struggled with questions that require critical thinking, stepwise analysis, and the application of logical reasoning. These higher-order cognitive skills are crucial for success in dental education and are assessed by the DAT. The results indicated the LLMs’ limitations for hallucinations and inaccuracies. LLMs can sometimes generate incorrect information, hallucinations, or unnecessary complexities, especially when dealing with intricate or complex questions. This could lead to students acquiring incorrect knowledge or misunderstandings.

## Discussion

The use of artificial intelligence (AI)-powered language models for test preparation represents an emerging and promising area in education technology. This study evaluated 16 LLMs, including commercial models, open-source models, and domain-specific fine-tuned models, to assess their ability to support preparation for the DAT. The quantitative and qualitative evaluation results highlight that while LLMs show potential in reinforcing factual knowledge and supporting personalized learning experiences, they also present significant risks in tasks that require higher-order cognitive skills such as critical analysis, synthesis, and evaluation. From a technical perspective, this study provides one of the most extensive evaluations of LLMs in a specialized educational domain, highlighting performance disparities across different model categories. For example, commercial models like GPT-4 and GPT-o1 excelled in text-based tasks, while fine-tuned domain-specific models (e.g., DentalGPT, MedGPT, and BioGPT) and open-source alternatives (e.g., Llama2 and Llama3 series) offered varied capabilities, with some demonstrating strong performance in areas like biology and chemistry [26,27]. This work extends prior research on general-purpose LLMs by incorporating fine-tuned and open-source models, as well as by addressing relatively underexplored areas such as the handling of image-based questions. The inclusion of image-based evaluations revealed critical areas for improvement, particularly in models’ ability to manage visual-spatial reasoning tasks. This finding underscores the limitations of current LLMs and provides valuable benchmarks for researchers and developers aiming to enhance educational applications [28]. Moreover, our results align with existing literature that highlights the utility of LLMs in providing factual knowledge and interactive tutoring capabilities that facilitate clarification and elaboration, which are especially beneficial for self-directed learners [26,27]. However, this study also emphasizes the importance of judiciously integrating LLMs with other resources, such as human instructors, study materials, and practice exams, to address their deficiencies in fostering critical thinking and advanced cognitive skills. Although AI tools have the potential to transform educational systems, they should not be used as standalone solutions, particularly for complex, high-stakes assessments like the DAT. One limitation of this study is the possibility that some DAT questions may have been included in the training data of the evaluated LLMs. While our findings suggest that this overlap is minimal, it cannot be entirely ruled out. Future studies should consider developing proprietary test datasets or adopting methods to ensure training data exclusion, thus enabling more robust and unbiased evaluations. Additionally, while the prompts used in this study were carefully designed to simulate the DAT context, we acknowledge that systematic experiments comparing different prompt designs were not conducted. Future studies could explore the impact of varying prompt phrasing and structure to better understand their influence on model performance and enhance the robustness of LLM evaluations.

From the quantitative results, all LLMs demonstrated strong capabilities in providing explanations and summaries of factual knowledge in areas such as biology, chemistry, and general science, which are critical for the DAT. This makes LLMs particularly valuable for learners seeking to acquire and reinforce factual knowledge. Additionally, the conversational mode of LLMs enables students to seek clarification or additional explanations on challenging topics, enhancing their understanding through interactive learning. Commercial models such as GPT-4 and GPT-o1 stood out with superior performance in text-based sections, with GPT-o1 achieving perfect accuracy in the NS and RC sections. Open-source models, such as Llama3-70B, also performed competitively, particularly in the RC section (100%), indicating that advancements in open-source architectures are narrowing the gap with proprietary models. Meanwhile, fine-tuned domain-specific models, such as DentalGPT and BioGPT, showed moderate success in the NS section but struggled in RC and QR tasks. This limitation likely stems from the reliance on general medical training datasets for fine-tuning, which may lack the specialized content necessary for the DAT, highlighting the importance of targeted fine-tuning for domain-specific tasks [29,30]. However, significant challenges were identified across all LLMs in image-based sections, particularly in the PA section. For instance, GPT-4o, despite its strong text-based performance, achieved only 24% accuracy in PA. Similarly, domain-specific models and open-source LLMs struggled in these tasks, with some (e.g., Llama series) not being evaluated due to architectural constraints. These results align with existing research that underscores the difficulty LLMs face in visual-spatial reasoning tasks [31]. Such findings emphasize the need for future advancements in multimodal capabilities to address these limitations effectively.

The thematic analysis highlighted possible drawbacks of using the LLMs in the DAT preparation. The accuracy and concordance of the model-generated text are difficult to guarantee. Most LLMs failed to effectively handle questions requiring critical thinking, stepwise reasoning, analysis, interpretation, and judgment. They often generated incorrect information, hallucinations, or unnecessary complexities. This is a common problem that still exists in LLMs [32–36]. The results underscore the superior performance of ChatGPT-4o in handling text-based questions across various sections of the DAT. However, the performance gap observed in image-based questions, particularly in the PA section, indicates an area for improvement for these models. The findings suggest that while current LLMs demonstrate proficiency in reinforcing factual knowledge through text, their ability to process and accurately respond to image-based content remains limited.

As LLM technologies continue to evolve, several directions emerge for enhancing their utility in educational settings. First, targeted fine-tuning with specialized datasets can improve domain-specific performance, as demonstrated by the moderate success of models like DentalGPT and BioGPT. Second, the integration of multimodal capabilities, enabling simultaneous text and image processing, could address current limitations in visual-spatial reasoning. Third, developing methods to mitigate hallucinations and ensure accuracy is essential for establishing trust and reliability in educational applications. Additionally, leveraging Biomedical Knowledge Graphs (BKGs) alongside Retrieval-Augmented Generation (RAG) systems offers promising strategies for improving LLM performance. BKGs provide structured and verified domain-specific knowledge, enhancing accuracy and contextual reasoning in complex queries, while RAG systems ensure that responses are grounded in external, up-to-date information [35–42]. Finally, longitudinal studies assessing the long-term impact of LLM-based tools on learners’ outcomes are needed. By comparing LLM-supported learning with traditional methods, future research can better understand the potential of AI in transforming education.

## Conclusion

This study provides valuable insights into the applications of LLMs in preparing for the DAT, demonstrating their strengths in reinforcing factual knowledge and their limitations in handling image-based and higher-order cognitive tasks. As AI technologies advance, their integration into educational systems must be approached judiciously, complementing traditional methods and addressing current shortcomings. The findings contribute to a growing body of research on LLMs in education, laying the groundwork for future innovations and applications.

## Ethical Approval

This study did not involve human participants or clinical trials; therefore, ethical approval was not required. The data used in this research consisted of publicly available sample questions from the dental admission test (DAT), obtained from the American Dental Association.

## Data Availability

The data used in this study, including the sample questions from the DAT, are publicly available and were sourced from the American Dental Association (https://www.ada.org/). No additional datasets were generated or analyzed during the study.
